# Spin-orbit engineering in transition metal dichalcogenide alloy monolayers

**DOI:** 10.1038/ncomms10110

**Published:** 2015-12-14

**Authors:** Gang Wang, Cedric Robert, Aslihan Suslu, Bin Chen, Sijie Yang, Sarah Alamdari, Iann C. Gerber, Thierry Amand, Xavier Marie, Sefaattin Tongay, Bernhard Urbaszek

**Affiliations:** 1 Université de Toulouse, INSA-CNRS-UPS, LPCNO, 135 Avenue Rangueil, 31077 Toulouse, France; 2 School for Engineering of Matter, Transport and Energy, Arizona State University, Tempe, Arizona 85287, USA

## Abstract

Binary transition metal dichalcogenide monolayers share common properties such as a direct optical bandgap, spin-orbit splittings of hundreds of meV, light–matter interaction dominated by robust excitons and coupled spin-valley states. Here we demonstrate spin-orbit-engineering in Mo_(1−*x*)_W_*x*_Se_2_ alloy monolayers for optoelectronics and applications based on spin- and valley-control. We probe the impact of the tuning of the conduction band spin-orbit spin-splitting on the bright versus dark exciton population. For MoSe_2_ monolayers, the photoluminescence intensity decreases as a function of temperature by an order of magnitude (4–300 K), whereas for WSe_2_ we measure surprisingly an order of magnitude increase. The ternary material shows a trend between these two extreme behaviours. We also show a non-linear increase of the valley polarization as a function of tungsten concentration, where 40% tungsten incorporation is sufficient to achieve valley polarization as high as in binary WSe_2_.

## Abstract (web summary)

Single atomic layers of transition metal dichalcogenides are semiconductors with possible applications in spintronics. Here, the authors demonstrate tuning of the spin-orbit splitting in molybdenum tungsten diselenide by altering the alloy's composition, impacting valley polarization and light emission yield.

## Graphical abstract

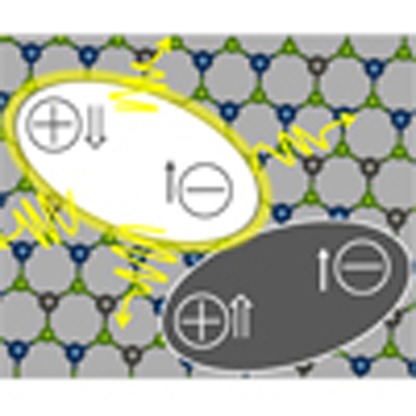

Transition metal dichalcogenide (TMDC) monolayers are newly discovered semiconductors for a wide range of applications in electronics and optoelectronics^1^,^2^,^3^,^4^. In addition to detailed studies on binary monolayers^5^,^6^,^7^,^8^,^9^,^10^,^11^, recent work on alloy-based monolayers have allowed tuning the optical bandgap^12^,^13^,^14^,^15^,^16^,^17^,^18^,^19^,^20^,^21^. The current generation of commercial optoelectronic devices based on III–V semiconductors owes its success to alloy engineering, adapting the optical and electronic properties of epitaxial layers by tuning the band structure and band alignment of each layer^22^. TMDC alloys provide additional tuning possibilities as strong spin-orbit (SO) coupling splits the spin states in the valence band (hundreds of meV)^3^,^23^ and in the conduction band (up to few tens of meV)^5^,^24^,^25^. Because the SO-coupling depends mainly on the transition metal d-orbitals, the valence band spin-splitting for WSe_2_ (∼400 meV (ref. 23)) is roughly twice that of MoSe_2_ (∼200 meV (ref. 3)). This should allow for a wide-tuning range in ternary alloys^13^. However, integrating monolayer (ML) TMDCs in realistic photonic and optoelectronic devices demands efficient optical emission.

For high optical quality alloy materials, the first challenge during crystal growth is to avoid phase separation and defect/cluster formation. This is a particular concern for materials containing anion atoms with large electronegativity or lattice constant differences. Owing to the rather close electronegativity and lattice constant values, as well as high miscibility, TMDCs alloys are stable at room temperatures^26^. Previously, TMDCs alloys were synthesized either by mixing different chalcogen atoms (MX_2(1−*n*)_Y_2*n*_) or transition metals (M_(1−*x*)_Z_*x*_X_2_) for broadly tunable optical bandgaps. This was achieved using chemical vapour deposition or conventional low-pressure vapour transport (LPVT) techniques to yield materials in monolayer or bulk (layered) form^13^,^18^,^19^,^14^.

In the following, we experimentally demonstrate efficient optical emission in a wide-tuning range in Mo_(1−*x*)_W_*x*_Se_2_. Here the sign of the spin-splitting in the conduction band, which depends on the transition metal^5^,^24^,^25^, determines the nature of the A-exciton ground state as optically bright or dark (Fig. 1a), where electron-hole Coulomb exchange effects also play a role^27^. Our experiments demonstrate that the light emission efficiency can be tuned by alloying. The sign of the spin-splitting has also important consequences for spin and valley control. By studying monolayers of high quality (Mo, W)Se_2_ ternary alloys at various compositions, we tune the SO-induced spin splittings, which strongly impact the optically generated valley polarization and the bright versus dark exciton recombination.

## Results

### Preparation and characterization of highly crystalline samples

**Figure 1:**
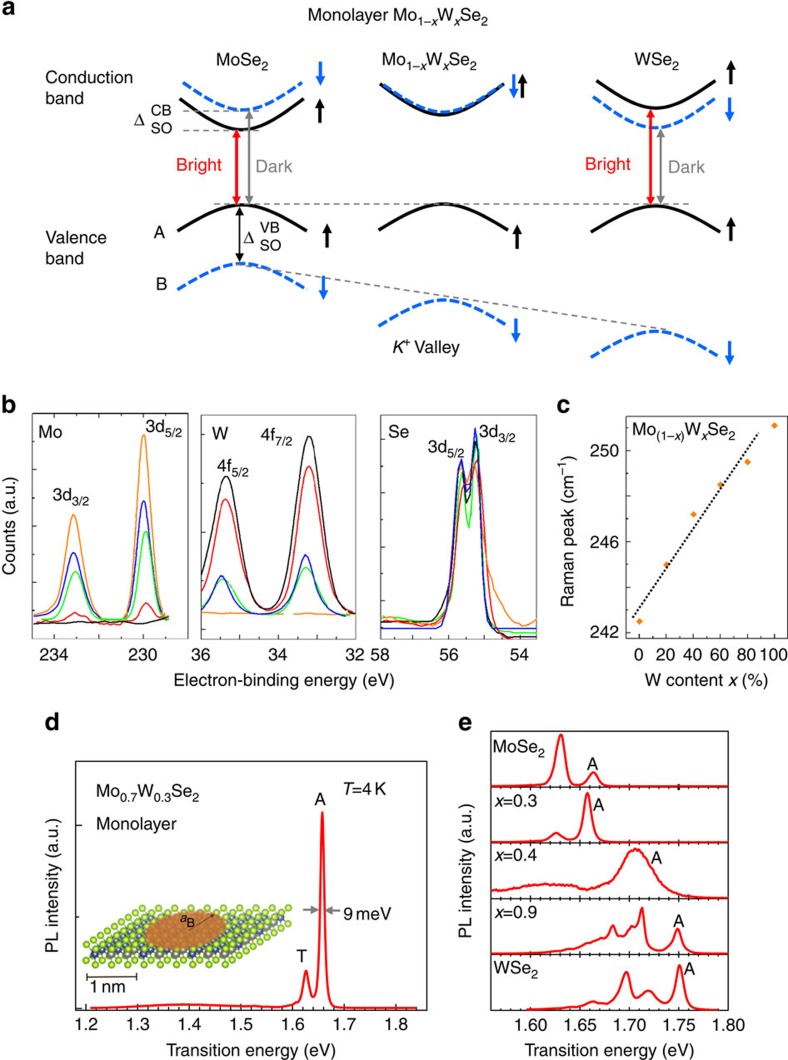
Highly crystalline Mo_(1−*x*)_W_*x*_Se_2_ alloy monolayers for spin-orbit engineering. (**a**) Simple band structure scheme in the *K*^+^ valley (at the *K* point of the Brillouin zone) to indicate the different signs and magnitudes of the valence 
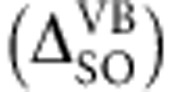
 and conduction band spin splittings 
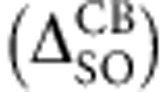
 when going from MoSe_2_ to WSe_2_ monolayers. Optically bright (red arrows) and dark (grey arrows) A-exciton transitions are indicated. (**b**) Nano-resolution x-ray photoelectron (nano-XPS) measurements on Mo_(1−*x*)_W_*x*_Se_2_ alloys showing gradual composition change with varying *x*, where orange, green, blue, red, black correspond to *x*=0%, 30%, 40%, 90 and 100%, respectively. For increasing *x* (W) content, W (Mo) content increases (decreases), whereas selenium ratio remains at the same values without any significant single or double (*V*_*Se*_ and *V*_2*Se*_) vacancy formation. (**c**) *E*_2*g*_ Raman peak position shift as a function of composition *x*. (**d**) Low temperature photoluminescence (PL) spectroscopy is a very simple and efficient technique to probe the material quality. Impurities and defects will trap optically excited carriers, resulting in emission below the optical bandgap. PL spectrum at *T*=4 K of Mo_0.7_W_0.3_Se_2_ alloy monolayer showing very sharp emission of the charged exciton (trion T) and the neutral A-exciton (A) and negligible defect-related emission. Inset: representation of the alloy monolayer, the order of magnitude of the Bohr radius *a*_B_ of an electron–hole pair (exciton) is shown. The narrow PL linewidth therefore indicates high quality alloy material on a nano-scopic scale. (**e**) PL spectra at 4 K of monolayers for tungsten (W) composition from *x*=0 to 100% in Mo_(1−*x*)_W_*x*_Se_2_. The dominant, sharp A-exciton emission is indicated.

For this study Mo_(1−*x*)_W_*x*_Se_2_ monolayers were exfoliated from their bulk counterparts, which were grown using LPVT (see Methods). We confirm the intended stoichiometry values using nano-x-ray photoelectron spectroscopy (nano-XPS), see Fig. 1b. Nano-XPS data taken from different spots closely match each other with 2% W/Mo deviation and implies that synthesized materials are uniform in composition. For different *x* values in Mo_(1−*x*)_W_*x*_Se_2_ monolayers, the most prominent in-plane Raman peak (E_2*g*_) gradually shifts from 241 to 251 cm^−1^ in Fig. 1c with slight deviation from linearity, which can be explained by the modified random element iso-displacement model^12^,^19^.

Photoluminescence (PL) results shown in Fig. 1d confirm the high quality of the ternary monolayers. Remarkably, in monolayer Mo_0.7_W_0.3_Se_2_, we detect virtually no defect-related emission. The PL is dominated by two sharp peaks, the neutral exciton *A* and the trion *T*, just as in the case of binary MoSe_2_ (refs 28, 29). The A-exciton PL full width at half maximum (FWHM) of only 9 meV is extremely narrow for a ternary alloy compared, for instance, with the typical PL FWHM of binary MoS_2_ of about 50 meV (ref. 4). The narrow PL linewidth allows us to determine the energy position of the A-exciton as a function of tungsten content in Fig. 1e. We observe band gap bowing as we deviate from a strictly linear extrapolation between the binary bandgaps^12^,^13^,^15^,^16^,^17^.

### Determination of A- to B-exciton splitting

**Figure 2:**
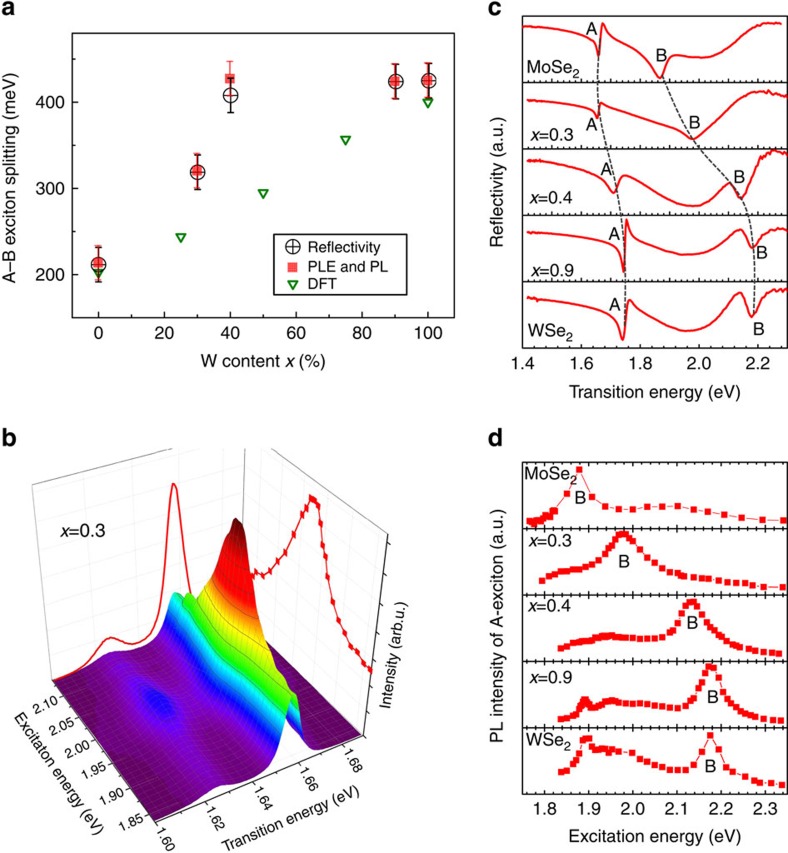
Tuning the spin-orbit splitting in Mo_(1−*x*)_W_*x*_Se_2_ monolayers. (**a**) The splitting between A- and B-excitons is measured by PL excitation spectroscopy (PLE) (red squares), where error bars correspond to laser energy step size and reflectivity (open circles), with error bars from multi-Lorentzian fits. The sum of the valence band and conduction band spin splittings calculated by DFT is shown for comparison, for individual values and computational details see Methods and Supplementary Note 1. (**b**) PL spectra of A-exciton in monolayer Mo_0.7_W_0.3_Se_2_ for different laser excitation energies. We uncover a clear maximum when the laser energy is in resonance with the B-exciton. (**c**) Reflectivity spectra using a white light source, uncovering in addition to the A-exciton also the B-exciton spectral position that can be tuned by varying the alloy composition. Dotted lines are a guide to the eye to indicate the energy shifts. (**d**) Same measurements as **b** but for all samples, the A-exciton PL intensity is plotted as a function of laser energy. These PLE measurements allow determining the B-exciton energy with very high precision.

Having analysed the high quality and alloy composition of the samples, we now aim to determine how the SO-coupling can be tuned for optoelectronics and valley polarization control. Reflectivity gives access to both A- and B-exciton energies, whose separation, determined mainly by SO-coupling, is plotted in Fig. 2a. Reflectivity spectra are shown in Fig. 2c. Spectrally narrow transitions confirm the position of the A-exciton at the same energy as the PL measurements for *x*=0.3 and *x*=0.9, indicating that exciton localization (Stokes shift) on defects, for example, is negligible. The transition several hundred meV above the A-exciton is due to the B-exciton that clearly shifts as the tungsten content is increased. We also perform PL excitation spectroscopy to access further details: When the laser is in resonance with the B-exciton, we observe a strong enhancement of the A-exciton PL emission intensity, as can be clearly seen for monolayer Mo_0.7_W_0.3_Se_2_ in Fig. 2b for a laser energy of 1.98 eV. We have performed experiments identical to Fig. 2b for all investigated samples using 30 different laser energies. The results are presented in Fig. 2d using the A-exciton as the detection energy. We clearly see a shift in energy of the maxima associated to the B-exciton as the tungsten content is varied, in agreement with our reflectivity results. Over the *x*=30–40% range, major changes of the exciton energies and SO splittings can be seen in our measurements. As a consequence, minor alloy fluctuations could lead to a spread in transition energies in this critical alloy composition range as for *x*=40% in Fig. 1e. This is not observed to this extent for other composition ranges.

The values of the A–B exciton splitting measured with two independent techniques are summarized in Fig. 2a. The valence band SO-splitting can be measured directly by angle-resolved photoemission spectroscopy (ARPES), for example, refs 3, 23. In optical spectroscopy, the energy position of the exciton transitions will in addition depend on the difference between A- and B-exciton-binding energies (expected to be small following measurements of similar values for 1s to 2s/2p separation for A- and B-excitons^8^,^30^) and the SO-splitting in the conduction band (up to tens of meV (refs 24, 25)). For Mo_0.7_W_0.3_Se_2_ monolayers, we measure an A–B exciton separation of 320 meV, an increase of 50% compared with binary MoSe_2_. For Mo_0.6_W_0.4_Se_2_, the separation is about 420 meV, very close to the value of 425 meV we measure for binary WSe_2_. Surprisingly, our experiments show a clear SO bowing, which has not been anticipated yet by theory as SO effects where not included in refs 15, 16, 17. Spin-orbit bowing has been observed in other systems such as III–V semiconductor alloys^31^. For order of magnitude estimations, we have calculated valence and conduction band splittings by density functional theory using an artificial supercell with just four transition metal atoms in ordered configurations, as shown in Supplementary Fig. 1 with details in Supplementary Note 1 and Supplementary Table 1. We show the sum of valence and conduction band splittings for comparison with the experiments in Fig. 2a, the influence of the realistic local lattice symmetry^15^,^16^,^17^,^31^, possibly different A- and B-exciton-binding energies and effective masses is not included^5^.

### Investigating order of dark and bright exciton states

**Figure 3:**
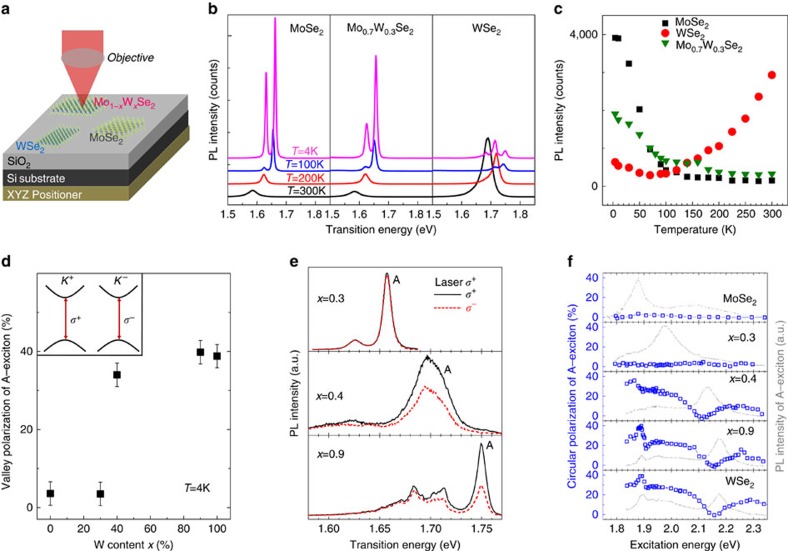
Temperature dependence and valley polarization engineering in Mo_(1−*x*)_W_*x*_Se_2_ monolayers. (**a**) Comparing the global PL emission intensity of several samples is challenging, as the set-up has to compensate thermal expansion/movement. For the measurements of the PL emission intensity as a function of temperature, we have exfoliated several monolayer flakes of different materials in close proximity onto the same substrate, which is mounted on a three-axis attocube nano-positioner. Therefore PL emission for different samples are measured under identical conditions, that is, same detection and laser spot size. Only highly reproducible in-plane movement is needed to change sample. (**b**) The PL spectra of monolayer MoSe_2_, Mo_0.3_W_0.7_Se_2_ and WSe_2_ are shown for different temperatures, the relative intensities can be directly compared. (**c**) Using the spectra from **b**, we integrate the total number of counts including A-exciton and trion (MoSe_2_ and Mo_0.3_W_0.7_Se_2_) and in addition the localized states (WSe_2_). We compare the total number of counts for the three monolayer materials as a function of temperature, see Supplementary Note 2 for details (**d**) The measured valley polarization, that is, circular PL polarization degree *P*_c_ is plotted as a function of tungsten (W) content in the sample. *P*_c_ is defined as 
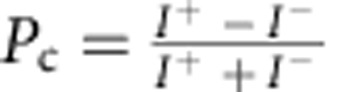
, where *I*^+^ and *I*^−^ are the *σ*^+^ and *σ*^−^ polarized PL components, respectively. We observe a highly non-linear increase in the valley polarization as more tungsten is incorporated. For the measurement, for each sample the laser energy is 140 meV above the A-exciton. The error bars correspond to the polarization resolution of our set-up. (**e**) Using *σ*^+^ circularly polarized laser excitation, we detect the A-exciton emission in *σ*^+^ (black) and *σ*^−^ (red) polarization. For *x*=0.3, we detect no polarization, as for binary MoSe_2_. Surprisingly, for *x*=0.4 we detect up to 40% PL polarization. The results for *x*=0.9 also show high polarization. (**f**) The circular PL polarization *P*_c_ is plotted as a function of the excitation laser energy to find optimal valley polarization conditions. While for *x*≤0.3 the valley polarization remains low, we demonstrate for *x*⩾0.4 a wide range of laser excitation energies that can be used for valley index initialization.

We have quantified the evolution of A–B exciton separation, mainly due to SO-coupling. Our next target is to show how tuning the amplitude and for the conduction band possibly the sign of the SO-coupling can be used to engineer the optoelectronic and spin-valley properties. Increasing the light emission efficiency is one of the main challenges for successful integration of TMDC MLs in realistic photonic and optoelectronic devices. This challenge can be tackled by SO-engineering in alloys, as the sign and amplitude of the SO-splitting in the conduction band will strongly influence the balance between optically dark and bright transitions in WSe_2_, MoSe_2_ and ternary alloys (see energy level diagram in Fig. 1a). This has a direct impact on the light emission yield from cryogenic to room temperatures, the SO-coupling therefore needs to be controlled for optoelectronics applications. Our set-up sketched in Fig. 3a allows for a quantitative comparison between samples. We are able to uncover important differences between WSe_2_, MoSe_2_ and the ternary samples, as can be seen directly when comparing the PL intensities in Fig. 3b. For monolayer WSe_2_, we detect comparatively weak emission at 4 K, increasing by 1 order of magnitude when going to *T*=300 K (Fig. 3c), a trend also observed in ref. 32. One possible explanation for the increase of the PL intensity as a function temperature is the thermal conversion of dark into bright states, as observed for CdSe nano-crystals^33^, where dark states also lie energetically below bright states. In stark contrast, for binary MoSe_2_ monolayers, we find a drastic decrease of the PL emission intensity when going from *T*=4 to 150 K, consistent with the opposite order of bright and dark states compared with WSe_2_, see Fig. 1a. The ternary sample Mo_0.3_W_0.7_Se_2_ shows a behaviour qualitatively similar to binary MoSe_2_, *albeit* with much stronger emission for temperatures from 100 to 200 K. For temperatures between 4 and 100 K, we are able to extract separately the PL intensity evolution of the trion and the neutral A-exciton for binary and ternary samples, see Supplementary Fig. 2 for full details. For binary MoSe_2_, we find that the trion intensity decreases much faster with temperature than the neutral exciton one. In contrast, for monolayer Mo_0.3_W_0.7_Se_2_ the trion and A-exciton PL intensity show similar temperature dependence. For a fully quantitative analysis of the global PL yield evolution, non-radiative channels due to material imperfections need to be taken into account in addition to the dark-bright state competition discussed here. We have confirmed the stark contrast between MoSe_2_ and WSe_2_ for monolayers not only from our LPVT samples but also from commercial material, which shows the same behaviour (see Supplementary Fig. 3). These temperature-dependent PL measurements provide a first experimental indication of the sign of the conduction band SO-splitting in these promising materials for optoelectronics. A detailed analysis of the potential impact of the bright versus dark exciton recombination in MoSe_2_ and WSe_2_ monolayers can be found in the very recent theory of ref. 34. For a more direct measurement of the conduction band spin splittings, ARPES experiments with sufficient energy resolution (meV range) would be desirable in future experiments.

### Probing optical valley initialization

We also investigate the impact of tuning the sign and amplitude of the SO-coupling on spin and valley physics in TMDC monolayers. The electron valley degree-of-freedom is accessible in monolayer TMDCs in simple optical manipulation schemes^2^,^10^,^11^ and is coupled to the electron spin degree-of-freedom. For this reason valleytronics is one of the main research directions in TMDCs^2^,^9^. Energetically degenerate states in the *K*^+^ and *K*^−^ valleys have opposite spins due to time reversal symmetry^2^,^10^. Large SO splittings will also stabilize the valley index, as spin flips are necessary to change valley in momentum space. Although MoSe_2_ and WSe_2_ show comparable structural and optical quality^28^,^35^,^36^, their valley polarization properties are very different: in WSe_2_ not only valley polarization can be generated, but also a superposition of *K*^+^ and *K*^−^ states, termed exciton valley coherence^35^. In contrast, the valley polarization of MoSe_2_ at zero magnetic field rarely exceeds 5% using various optical initialization schemes^29^,^36^. Here we investigate how valley polarization generation improves in TMDC alloys as we go from MoSe_2_ to WSe_2_, see Fig. 3d. For the high quality *x*=0.3 sample, we do not detect measurable valley polarization when scanning a very broad range of laser energies, as can be seen in Fig. 3e,f. By increasing the tungsten content by only 10% to *x*=0.4 we can increase the valley polarization by an order of magnitude to about 40%. An efficient way for successful optical valley initialization of the A-exciton is to tune the excitation laser into resonance with exciton ground or excited states^8^. For MoSe_2_, the B-exciton 1s and the A-exciton 2s/2p states overlap energetically^30^, which might be one of the reasons that prevents optical valley initialization. The specific band structure of ML MoSe_2_ could also play a role^30^. For WSe_2_, the situation is much simpler, as the B-excion is at much higher energy compared with the excited A-exciton states. For *x*=0.4, we are again in this favourable situation, which might be beneficial for optical valley initialization.

In summary, we demonstrate SO engineering in Mo_(1−*x*)_W_*x*_Se_2_ alloy monolayers for optoelectronics and applications based on electron spin- and valley-control. We show a non-linear increase of the optically generated valley polarization as a function of tungsten concentration, where 40% tungsten incorporation is sufficient to achieve valley polarization as high as in binary WSe_2_. We also probe the impact of the tuning of the conduction band SO spin-splitting on the bright versus dark exciton population, that is, PL emission intensity. We show that the MoSe_2_ PL intensity decreases as a function of temperature by an order of magnitude, whereas for WSe_2_ we measure surprisingly an order of magnitude increase over the same temperature range (*T*=4–300 K). The ternary material shows a trend between these two extreme behaviours. These results demonstrate that alloying is a promising technique for optimizing the light emission efficiency and valley index stability in two-dimensional TMDCs.

## Methods

### Sample growth and characterization

Layered Mo_(1−*x*)_W_*x*_Se_2_ semiconductors were grown by modified LPVT technique to achieve high optical quality materials. Synthesized materials are fully alloyed (not phase separated) as confirmed by three complementary techniques, such as sub-micron Raman spectroscopy, sub-micron PL and nano-XPS, see Fig. 1. X-ray diffraction measurements display sharp (∼1-μm domain size) (001) peaks. Thus TMDCs alloys are highly layered and crystallized in the hexagonal phase, and contains negligible amount of minority crystal orientation. Observed (001) peaks of Mo_(1−*x*)_W_*x*_Se_2_ appear at the same position (within <0.08°) implying that alloys have similar c-axis parameters. Consistent with nano-XPS measurements, Rutherford backscattering spectroscopy data collected from ∼100-μm diameter circle agrees well with the XPS measurements (not shown).

### Optical spectroscopy

Experiments at *T*=4–300 K are carried out in a confocal microscope^8^. The detection spot diameter is ≈1 μm, that is, considerably smaller than the monolayer flake size of ∼10 × 10 μm^2^. For time integrated experiments, the PL emission is dispersed in a spectrometer and detected with a Si-CCD camera. The samples are excited either by a continuous wave (cw) He–Ne laser or pico-second (ps) pulses generated by a tunable frequency-doubled optical parametric oscillator synchronously pumped by a mode-locked Ti:Sa laser. The typical pulse temporal and spectral widths are 1.6 ps and 3 meV, respectively, the repetition rate is 80 MHz.

### Theory

The electronic structures of Mo_(1−*x*)_W_*x*_Se_2_ with *x*=0.25,0.50,0.75 were simulated within the DFT framework as implemented in the VASP^37^ package. The conduction and valence spin-splitting of these elemental compositions were simply computed in a (2 × 2) supercell, containing four transition metal atoms in total and neglecting for simplicity random and more realistic configurations, see Supplementary Fig. 1 and Supplementary Note 1.

## Additional information

**How to cite this article:** Wang, G. *et al*. Spin-orbit engineering in transition metal dichalcogenide alloy monolayers. *Nat. Commun.* 6:10110 doi: 10.1038/ncomms10110 (2015).

## Supplementary Material

Supplementary Information Supplementary Figures 1-3, Supplementary Table 1, Supplementary Notes 1-2 and Supplementary References

## References

[b1] Lopez-Sanchez O., Lembke D., Kayci M., Radenovic A. & Kis A. Ultrasensitive photodetectors based on monolayer mos2. Nat. Nanotechnol. 8, 497501 (2013). 10.1038/nnano.2013.100 23748194

[b2] Xu X., Xiao D., Heinz T. F. & Yao W. Spin and pseudospins in layered transition metal dichalcogenides. Nat. Phys. 10, 343–350 (2014).

[b3] Zhang Y. . Direct observation of the transition from indirect to direct bandgap in atomically thin epitaxial mose2. Nat. Nanotechnol. 9, 111–115 (2014). 24362235 10.1038/nnano.2013.277

[b4] Mak K. F., Lee C., Hone J., Shan J. & Heinz T. F. Atomically thin mos_2_: a new direct-gap semiconductor. Phys. Rev. Lett. 105, 136805 (2010). 21230799 10.1103/PhysRevLett.105.136805

[b5] Kormanyos A. . k.p theory for two-dimensional transition metal dichalcogenide semiconductors. 2D Mater. 2, 022001 (2015).

[b6] Ye Z. . Probing excitonic dark states in single-layer tungsten disulfide. Nature 513, 214–218 (2014). 25162523 10.1038/nature13734

[b7] Ugeda M. M. . Observation of giant bandgap renormalization and excitonic effects in a monolayer transition metal dichalcogenide semiconductor. Nat. Mater. 13, 1091–1095 (2014). 25173579 10.1038/nmat4061

[b8] Wang G. . Giant enhancement of the optical second-harmonic emission of wse_2_ monolayers by laser excitation at exciton resonances. Phys. Rev. Lett. 114, 097403 (2015). 25793850 10.1103/PhysRevLett.114.097403

[b9] Mak K. F., McGill K. L., Park J. & McEuen P. L. The valley hall effect in mos2 transistors. Science 344, 1489–1492 (2014). 24970080 10.1126/science.1250140

[b10] Cao T. . Valley-selective circular dichroism in mos_2_. Nat. Commun. 3, 887 (2012). 22673914 10.1038/ncomms1882 PMC3621397

[b11] Sallen G. . Robust optical emission polarization in mos_2_ monolayers through selective valley excitation. Phys. Rev. B 86, 081301 (2012).

[b12] Tongay S. . Two-dimensional semiconductor alloys: Monolayer mo1xwxse2. Appl. Phys. Lett. 104, 012101 (2014).

[b13] Chen Y. . Tunable band gap photoluminescence from atomically thin transition-metal dichalcogenide alloys. ACS Nano 7, 4610–4616 (2013). 23600688 10.1021/nn401420h

[b14] Zheng S. . Monolayers of wxmo1xs2 alloy heterostructure with in-plane composition variations. Appl. Phys. Lett. 106, 063113 (2015).

[b15] Kutana A., Penev E. S. & Yakobson B. I. Engineering electronic properties of layered transition-metal dichalcogenide compounds through alloying. Nanoscale 6, 5820–5825 (2014). 24744083 10.1039/c4nr00177j

[b16] Gan L.-Y., Zhang Q., Zhao Y.-J., Cheng Y. & Schwingenschlögl U. Order-disorder phase transitions in the two-dimensional semiconducting transition metal dichalcogenide alloys Mo_1−*x*_W_*x*_X_2_ (X=S, Se, and Te). Sci. Rep. 4, 6691–6695 (2014). 25331363 10.1038/srep06691 PMC4204064

[b17] Xi J., Zhao T., Wang D. & Shuai Z. Tunable electronic properties of two-dimensional transition metal dichalcogenide alloys: a first-principles prediction. J. Phys. Chem. Lett. 5, 285–291 (2014). 26270701 10.1021/jz402375s

[b18] Feng Q. . Growth of large-area 2d mos2(1-x)se2x semiconductor alloys. Adv. Mater. 26, 2648–2653 (2014). 24677312 10.1002/adma.201306095

[b19] Chen Y. . Composition-dependent raman modes of mo1-xwxs2 monolayer alloys. Nanoscale 6, 2833–2839 (2014). 24469100 10.1039/c3nr05630a

[b20] Zhang M. . Two-dimensional molybdenum tungsten diselenide alloys: Photoluminescence, raman scattering, and electrical transport. ACS Nano 8, 7130–7137 (2014). 24884059 10.1021/nn5020566

[b21] Su S.-H. . Band gap-tunable molybdenum sulfide selenide monolayer alloy. Small 10, 2589–2594 (2014). 24610642 10.1002/smll.201302893

[b22] Rosencher E. & Vinter B. Optoelectronics Cambridge Univ. Press (2002).

[b23] Riley J. . Direct observation of spin-polarized bulk bands in an inversion-symmetric semiconductor. Nat. Phys. 10, 835–839 (2014).

[b24] Liu G.-B., Shan W.-Y., Yao Y., Yao W. & Xiao D. Three-band tight-binding model for monolayers of group-vib transition metal dichalcogenides. Phys. Rev. B 88, 085433 (2013).

[b25] Kosmider K., González J. W. & Fernández-Rossier J. Large spin splitting in the conduction band of transition metal dichalcogenide monolayers. Phys. Rev. B 88, 245436 (2013).

[b26] Kang J., Tongay S., Li J. & Wu J. Monolayer semiconducting transition metal dichalcogenide alloys: Stability and band bowing. J. Appl. Phys. 113, 143703 (2013).

[b27] Qiu D. Y., Cao T. & Louie S. G. Nonanalyticity, valley quantum phases, and lightlike exciton dispersion in monolayer transition metal dichalcogenides: Theory and first-principles calculations. Phys. Rev. Lett. 115, 176801 (2015). 26551134 10.1103/PhysRevLett.115.176801

[b28] Ross J. S. . Electrical control of neutral and charged excitons in a monolayer semiconductor. Nat. Commun. 4, 1474 (2013). 23403575 10.1038/ncomms2498

[b29] Wang G. . Polarization and time-resolved photoluminescence spectroscopy of excitons in mose2 monolayers. Appl. Phys. Lett. 106, 112101 (2015).

[b30] Wang G. . Exciton states in monolayer MoSe2: impact on interband transitions. 2D Mater 2, 045005 (2015).

[b31] Wei S.-H. & Zunger A. Band gaps and spin-orbit splitting of ordered and disordered al_*x*_ga_1−*x*_As and Gaas_*x*_sb_1−*x*_ alloys. Phys. Rev. B 39, 3279–3304 (1989). 10.1103/physrevb.39.3279 9948630

[b32] Arora A. . Excitonic resonances in thin films of WSe2: From monolayer to bulk material. Nanoscale 7, 10421–10429 (2015). 25998778 10.1039/c5nr01536g

[b33] Crooker S. A., Barrick T., Hollingsworth J. A. & Klimov V. I. Multiple temperature regimes of radiative decay in cdse nanocrystal quantum dots: Intrinsic limits to the dark-exciton lifetime. Appl. Phys. Lett. 82, 2793–2795 (2003).

[b34] Dery H. & Song Y. Polarization analysis of excitons in monolayer and bilayer transition-metal dichalcogenides. Phys. Rev. B 92, 125431 (2015).

[b35] Jones A. M. . Optical generation of excitonic valley coherence in monolayer wse2. Nat. Nanotechnol. 8, 634–638 (2013). 23934096 10.1038/nnano.2013.151

[b36] MacNeill D. . Breaking of valley degeneracy by magnetic field in monolayer mose_2_. Phys. Rev. Lett. 114, 037401 (2015). 25659021 10.1103/PhysRevLett.114.037401

[b37] Kresse G. & Furthmüller J. Efficient iterative schemes for *ab initio* total-energy calculations using a plane-wave basis set. Phys. Rev. B 54, 11169–11186 (1996). 10.1103/physrevb.54.11169 9984901

