# Supplementary material for: Spin-orbit engineering in transition metal dichalcogenide alloy monolayers
Source: Nat Commun. 2015 Dec 14;6:10110. doi: 10.1038/ncomms10110 (PMC4682039; doi:10.1038/ncomms10110)
Supplement: Supplementary Information — Supplementary Figures 1-3, Supplementary Table 1, Supplementary Notes 1-2 and Supplementary References [file ncomms10110-s1.pdf]

a) b)

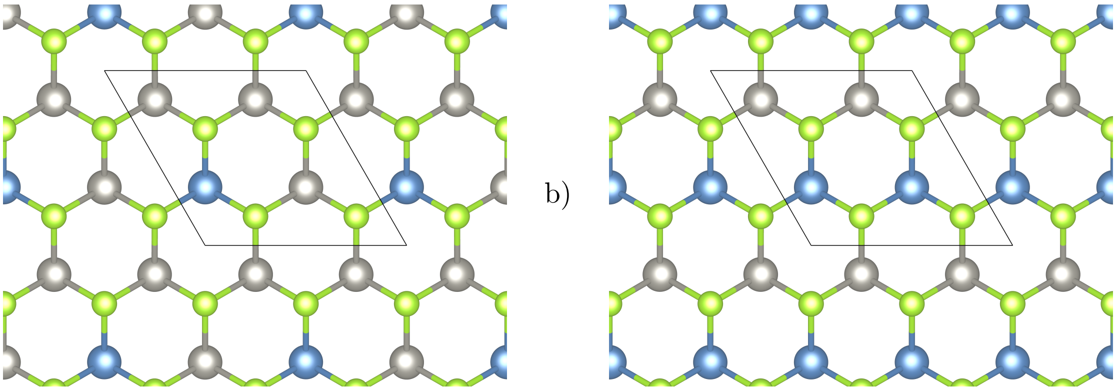

Supplementary Figure 1: Ordered configurations used for the calculations with (a) x=0.25 and (b) x=0.5. The calculation cell is given with the black line. Mo atoms are in blue, W atoms in grey and Se in green

.

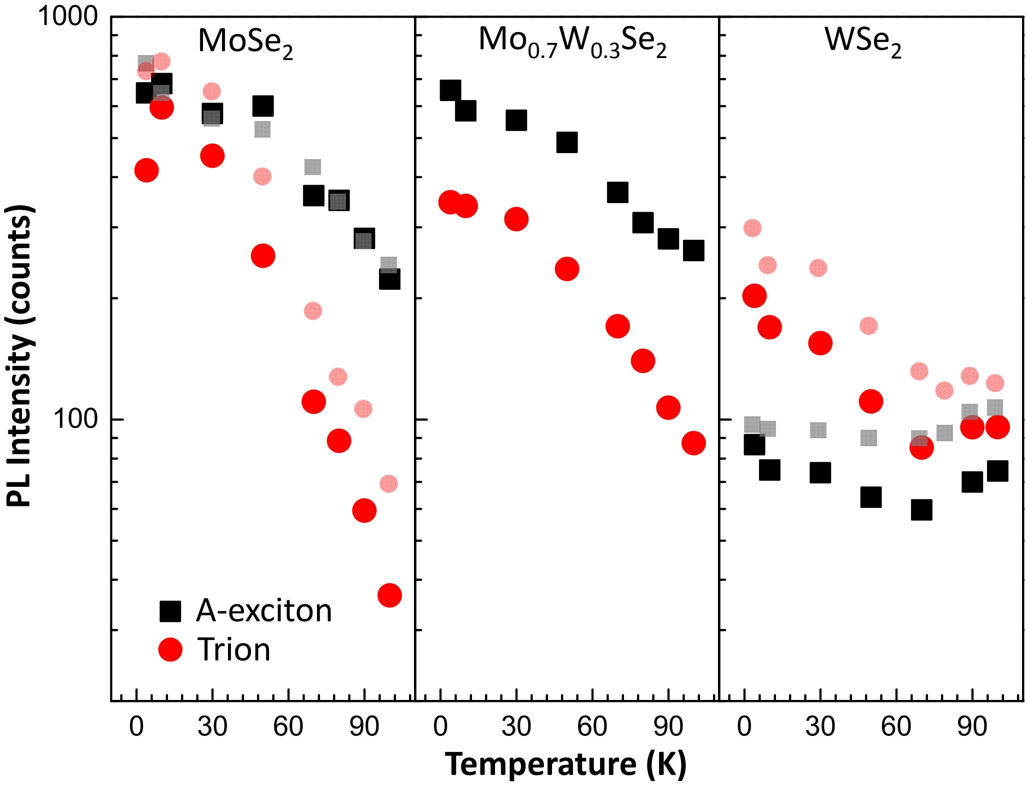

Supplementary Figure 2: Evolution with temperature of the neutral A-exction (black squares) and trion(red circles) PL emission intensity for ML $MoSe_2$, $Mo_{0.7}W_{0.3}Se_2$ and WSe_2. Bold, large symbols correspond to experiments on our LPVT grown samples, the smaller, semitransparent symbols show for comparison measurements on commercial (2D Semiconductors) binary material.

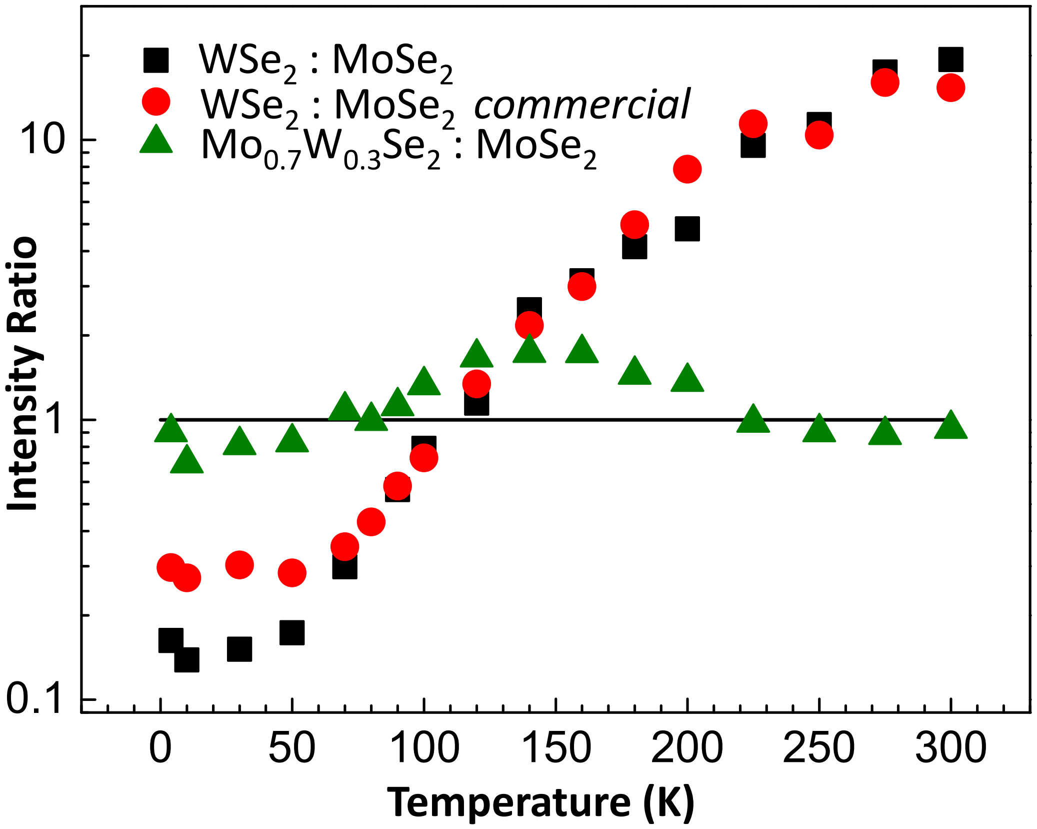

Supplementary Figure 3: PL emission intensity of three W containing samples is divided by the $MoSe_2$ PL intensity. The interest in plotting the ratio at each temperature is to eliminate any difference in detection efficiency that exist for different set-up temperatures. Commercial WSe_2 samples (red circles) show the same trend as our LPVT grown samples (black squares). Alloy $Mo_{0.3}W_{0.7}Se_2$: $MoSe_2$ PL ratio shown as green triangles.

	Exp. Mo-cell 3.289 Å		Exp. W-cell 3.282 Å	
x	Δ_{SO}^{VB}	Δ_{SO}^{CB}	Δ_{SO}^{VB}	Δ_{SO}^{CB}
0.0	452	+46	449	+49
0.25	375	+7	372	+15
0.5	305	+10	304	+9
0.75	242	-5	241	-3
1.0	182	-22	181	-22

Supplementary Table 1: Spin splitting energy values in the valence and the conduction bands in meV as function of the lattice parameter.

Supplementary Note 1 Density Functional Theory

Computational Details. First-principles calculations are performed using the Vienna Ab-initio Simulation Package [1–4] and the Perdew-Burke-Ernzerhof generalized gradient approximation [5] for exchange-correlation functional. It uses the plane-augmented wave scheme [6, 7] to treat core electrons. Fourteen and six electrons for metals and Se respectively are explicitly included in the valence, with an energy cut-off of 400 eV. All atoms are allowed to relax with a force convergence criterion below 0.005 eV/Å, using experimental lattice parameters of 3.289 and 3.282 Å for the $MoSe_2$-based and for WSe_2-based cell respectively. Spin-Orbit Coupling (SOC) is included non-self consistently, since for these calculations the valence band splitting deviates by less than 1 meV from the values obtained from self-consistent SOC calculations on primitive cells. A grid of 12×12×1 and 6×6×1 k-points have been used for primitive and (2×2) cell respectively, in conjunction with a

vacuum height of 17 Å. A gaussian smearing with a width of 0.05 eV is used for partial occupancies, when a tight electronic minimization tolerance of 10^{-8} eV.

Configurations. Random configurations in the lattice of $Mo_xW_{(1-x)}Se_2$ alloys will have an impact on the experiments in the present study and on previous ones for $Mo_xW_{(1-x)}S_2$ [8, 9] In our simple approach, the determination of the spin-splitting due to Spin-Orbit Coupling (SOC) in Conduction Band (CB) and Valence Band (VB) is only possible for ordered structures since supercell calculations suffer from band folding in the first Brillouin zone. Energetic aspects have theoretically studied in details for disulphide compounds mainly, but Gan *et al* have reported, based on DFT calculations, that Mo-Se-W bonds are slightly lower in energy than Mo-Se-Mo or W-Se-W bonds [10], a statement also confirmed by another work [11]. Besides in ref.[12], it is shown that when the percentage of W is increased the hole effective mass decreases linearly and more importantly the electron effective mass of alloys is always larger than that of the pure systems. Since transition metal d-orbitals contribute differently to conduction bands for MoS_2 and WS_2, but do not to valence bands, it is clear that electrons and holes in alloys behave differently when compared to pure systems. Considering band gaps, a slight bowing has been also reported at the LDA level, up to a W concentration of 0.5 [13]. In the present study, we have considered only ordered configurations based on a (2x2) supercell, thus neglecting the randomization effects of the site occupations. Supplementary Figure 1 shows these ordered atomic configurations for $x = 0.25$ and $x = 0.5$ that have been used to extract spin splitting energy values in the valence and the conduction bands for two different values of lattice parameter.

Lattice parameter effects. Influence of the lattice parameter choices on the spin-splitting of valence and conduction bands are presented in Supplementary Table 1. Using a slightly larger lattice parameter, i.e the Mo-cell one, provides larger spin-splitting energy in the valence bands, but smaller ones in the conduction bands. All together, the separation of the A and B exciton energies is mainly governed by the spin-splitting, in addition different effective masses and exciton binding energies can contribute.

Supplementary Note 2 Optical Spectroscopy

We analyse separately the evolution with temperature of the neutral A-exction and trion PL emission intensity by careful fitting multi-Gaussian lineshapes for ML $MoSe_2$,

$Mo_{0.7}W_{0.3}Se_2$ and WSe_2. Here we concentrate on the temperature range where trions and excitons can be clearly distinguished. The bold, large symbols correspond to experiments on our LPVT grown samples, the smaller, semitransparent symbols show for comparison measurements on commercial (2D Semiconductors) binary material. Note that the A-exciton to trion intensity ratio for each sample will depend on the excess carrier concentration. In our samples the residual doping level cannot be controlled, for more deterministic charge control gated samples are needed [14].

To go further in our direct comparison of PL emission intensities for different samples, we plot in Supplementary Figure 3 the ratio of the PL intensity of WSe_2 divided by the intensity of $MoSe_2$ measured during the same experiment. We observe for our LPVT grown samples and commercial samples the same trend. The $MoSe_2$ emission gets weaker as temperature increases, whereas WSe_2 emission intensity gets stronger. Very similar results for samples grown under different conditions indicate that the PL evolution is due to intrinsic effects such as the competition between spin-orbit split bright and dark states [15], and cannot merely be attributed to different sample quality. For the ternary sample we see a clear difference to the $MoSe_2$ evolution between $T = 100$ K and 200 K.

Supplementary References

[1] Kresse, G. & Hafner, J. *Ab initio* molecular dynamics for liquid metals. *Phys. Rev. B* **47**, 558–561 (1993).

[2] Kresse, G. & Hafner, J. *Ab initio* molecular-dynamics simulation of the liquid-metal–amorphous-semiconductor transition in germanium. *Physical Review B* **49**, 14251–14269 (1994).

[3] Kresse, G. & Furthmüller, J. Efficient iterative schemes for *ab initio* total-energy calculations using a plane-wave basis set. *Phys. Rev. B* **54**, 11169–11186 (1996).

[4] Kresse, G. & Furthmüller, J. *Comput. Mater. Sci.* **6**, 15 (1996).

[5] Perdew, J. P., Burke, K. & Ernzerhof, M. Generalized gradient approximation made simple. *Phys. Rev. Lett.* **77**, 3865–3868 (1996).

[6] Blöchl, P. E. Projector augmented-wave method. *Phys. Rev. B* **50**, 17953 (1994).

[7] Kresse, G. & Joubert, D. From ultrasoft pseudopotentials to the projector augmented-wave method. *Physical Review B* **59**, 1758–1775 (1999).

[8] Dumcenco, D. O., Kobayashi, H., Liu, Z., Huang, Y.-S. & Suenaga, K. Visualization and quantification of transition metal atomic mixing in $Mo_{1-x}W_xS_2$ single layers. *Nature Communications* **4**, 1351–5 (2012).

[9] Chen, Y. *et al.* Tunable band gap photoluminescence from atomically thin transition-metal dichalcogenide alloys. *ACS Nano* **7**, 4610–4616 (2013).

[10] Gan, L.-Y., Zhang, Q., Zhao, Y.-J., Cheng, Y. & Schwingenschlögl, U. Order-disorder phase transitions in the two-dimensional semiconducting transition metal dichalcogenide alloys $Mo_{1-x}W_xX_2$ (X = S, Se, and Te). *Scientific Reports* **4**, 6691–5 (2014).

[11] Wei, X.-L. *et al.* Modulating the atomic and electronic structures through alloying and heterostructure of single-layer MoS_2. *J. Mater. Chem. A* **2**, 2101–2109 (2014).

[12] Xi, J., Zhao, T., Wang, D. & Shuai, Z. Tunable Electronic Properties of Two-Dimensional Transition Metal Dichalcogenide Alloys: A First-Principles Prediction. *The Journal of Physical Chemistry Letters* **5**, 285–291 (2014).

[13] Kutana, A., Penev, E. S. & Yakobson, B. I. Engineering electronic properties of layered transition-metal dichalcogenide compounds through alloying. *Nanoscale* **6**, 5820–5825 (2014).

[14] Ross, J. S. *et al.* Electrical control of neutral and charged excitons in a monolayer semiconductor. *Nature communications* **4**, 1474 (2013).

[15] Dery, H. & Song, Y. Polarization analysis of excitons in monolayer and bilayer transition-metal dichalcogenides. *Phys. Rev. B* **92**, 125431 (2015).
